# Assessment of the Association Between Whole Blood Viscosity and Coronary Artery Calcium Score

**DOI:** 10.3390/medicina62010169

**Published:** 2026-01-14

**Authors:** Serkan Duyuler, Pınar Türker Duyuler, Süleyman Kalaycı, Koray Arslan, Raif Can Karabulut, Mustafa Dağlı

**Affiliations:** 1 Cardiology Department, İzmir City Hospital, 35530 İzmir, Turkey; 2 Cardiology Department, Bilkent City Hospital, 06800 Ankara, Turkey; dr_suleyman_kalayci@yahoo.com (S.K.); kryarslan1994@gmail.com (K.A.); karabulutraifcan@gmail.com (R.C.K.); 3 Radiology Department, Bilkent City Hospital, 06800 Ankara, Turkey; mdagli8033@gmail.com

**Keywords:** whole blood viscosity, coronary artery calcium score

## Abstract

*Background and Objectives*: Whole Blood Viscosity (WBV), estimated using the De Simone formula, is a key hemodynamic parameter linked to endothelial dysfunction and atherosclerosis. Its association with significant coronary calcification, defined as a high Coronary Artery Calcium Score (CACS ≥ 100), remains unclear. This study investigated whether calculated WBV predicts high CACS. *Materials and Methods*: In this single-center, retrospective, cross-sectional study, 403 patients undergoing coronary computed tomography angiography for suspected stable coronary artery disease were included. Participants were stratified into CACS < 100 (*n* = 258) and CACS ≥ 100 (*n* = 145). WBV was calculated at High Shear Rate (HSR) and Low Shear Rate (LSR) using the De Simone formula. Multivariate binomial logistic regression adjusted for conventional cardiovascular risk factors was used to identify independent predictors of high CACS. *Results*: Patients with CACS ≥ 100 were older, more frequently male, and had a higher prevalence of diabetes and hypertension (all *p* < 0.01). Mean WBV did not differ significantly between groups: WBV-HSR, 4.3 ± 0.5 cP vs. 4.4 ± 0.5 cP (*p* = 0.456); WBV-LSR, 29.9 ± 8.0 cP vs. 30.4 ± 8.6 cP (*p* = 0.505). In multivariate models, neither WBV-HSR (OR: 0.489; *p* = 0.462) nor WBV-LSR (OR: 0.987; *p* = 0.520) independently predicted high CACS. Age and sex were the strongest independent predictors (*p* < 0.001). *Conclusions*: No independent association was found between calculated WBV and high CACS in this cross-sectional study.

## 1. Introduction

Cardiovascular disease remains a leading cause of morbidity and mortality worldwide. Atherosclerosis is a central pathophysiological process underlying these conditions, and in addition to traditional risk factors, disturbances in blood rheology play a meaningful role in its development [1]. WBV is a critical hemodynamic variable that reflects the resistance of blood to flow. This resistance is mainly determined by the cellular content (hematocrit) and the protein composition of the plasma, both of which govern the friction and aggregation properties of blood. Elevated WBV increases shear stress on the vascular endothelium, fostering endothelial dysfunction—a pivotal mechanism that accelerates atherosclerotic progression [2]. The De Simone formula simplified the measurement of WBV, moving it from a specialized, complex laboratory test to a straightforward calculation based on routine clinical laboratory data [3].

A growing body of evidence supports the association between calculated WBV and multiple manifestations of atherosclerosis and Coronary Artery Disease (CAD). Increases in WBV are related to the carotid artery plaque, which reflects the earlier association with atherogenesis, and to stent restenosis, an indicator of very late manifestation [4,5]. Additionally, elevated WBV is independently associated with various forms of cardiac calcification, including Mitral Annular Calcification and Aortic Valve Sclerosis [6,7]. However, despite Coronary Artery Calcium Score (CACS)—a robust measure of calcified plaque burden—being highly prognostic, the direct relationship between WBV and CACS remains limited in the current literature.

While direct rheological measurements using a viscometer remain the gold standard, calculated whole blood viscosity (WBV) via the De Simone formula, which is primarily driven by hematocrit and total plasma protein, serves as a validated and clinically accessible surrogate for whole blood viscosity. On the other hand, while direct rheological measurements using a viscometer provide instantaneous physical properties of the blood, the De Simone formula integrates long-term determinants like hematocrit, which might be more reflective of the cumulative atherosclerotic process leading to calcification. The rationale for investigating the association between calculated WBV and coronary calcification is supported by recent evidence highlighting the role of its components in atherosclerosis. For instance, hematocrit has been identified as an independent risk factor for coronary heart disease (CHD) in patients with chest pain and has been shown to predict subclinical atherosclerosis, such as carotid intima-media thickness [8,9]. Furthermore, hemorheological alterations, including increased whole blood viscosity and hematocrit, have been documented in patients with CAD detected by multi-slice CT [10], suggesting a potential link between blood flow dynamics and anatomical plaque burden. Given that CACS is a strong predictor of cardiovascular events independent of traditional risk factors, and that WBV plays a central role in the pathogenesis of atherosclerosis, a positive correlation between WBV and CACS would be biologically plausible. Therefore, in this study, we aimed to investigate whether WBV, calculated using the De Simone formula, is independently associated with CACS as assessed by Coronary Computed Tomography Angiography (CCTA).

## 2. Materials and Methods

This study was designed as a single-center, retrospective, cross-sectional analysis. The required sample size was determined a priori using G*Power software (version 3.1.9.7). The calculation utilized a two-tailed binomial logistic regression model to predict high CACS (≥100) in patients. Based on established hemorheological literature, we aimed to detect a small-to-medium effect size (w = 0.2) with an alpha level of 0.05 and a statistical power (1 − β) of 0.80. This analysis indicated a minimum requirement of 341 patients. With a final study cohort of 403 participants, the study was deemed adequately powered to test the primary hypothesis. Furthermore, the actual power achieved in the final analysis was assessed. Using the observed effect size, N = 403 participants, and α = 0.05, the post hoc power (1 − β) reached 0.87, confirming the statistical robustness of the study’s conclusions. All participants were admitted to the Cardiology Clinic of Ankara Bilkent City Hospital between April 2023 and April 2025 and underwent CCTA due to a clinical suspicion of stable CAD. Typical indications included atypical chest pain, borderline findings on noninvasive stress testing, or a low-to-intermediate pre-test probability of CAD.

Patients aged ≥18 years with complete CCTA imaging and standard laboratory data were eligible. To preserve the accuracy of WBV estimation and cardiac measurements, individuals with conditions known to affect these parameters were excluded. These included a history of Myocardial Infarction (MI), Percutaneous Coronary Intervention (PCI), Coronary Artery Bypass Grafting (CABG), recent surgery or acute blood loss, chronic kidney or liver disease, paraproteinemia, hematologic disorders, or anemia.

Demographic characteristics, conventional cardiovascular risk factors, and laboratory measurements were obtained through retrospective review of hospital records. Hypertension was defined as a documented blood pressure ≥ 140/90 mmHg or the use of antihypertensive medication. Diabetes Mellitus was defined as a fasting plasma glucose level≥ 126 mg/dL or treatment with antidiabetic agents.

Fasting blood samples were analyzed using standard commercial laboratory kits to obtain hematologic parameters, including Hematocrit (Hct), and biochemical parameters, including Total Plasma Protein (TP). The WBV was calculated using the validated De Simone formula, which incorporates readily available Hct and TP values [3]. WBV was derived for both Low Shear Rate (LSR; 0.5 s^−1^) and High Shear Rate (HSR; 208 s^−1^) conditions and expressed in centipoise (cP) or millipascal-seconds (mPa·s). For consistency with prior literature, TP was expressed in g/dL and Hct as a percentage:

High Shear Rate (HSR) Formula:

WBV (208 s^−1^) = (0.12 × Hct) + 0.17 × TP g/dL − 2.07

Low Shear Rate (LSR) Formula:

WBV (0.5 s^−1^) = (1.89 × Hct) + 3.76 × TP g/dL − 78.42

Coronary artery calcification was assessed using a 512-row Multi-Detector Computed Tomography (MDCT) scanner (Revolution; GE Healthcare, Milwaukee, WI, USA) with a standard non-contrast protocol. Calcification severity was quantified using the validated Agatston scoring method. Patients were stratified into two groups according to established clinical thresholds: CACS < 100 and CACS ≥ 100, the latter representing a widely accepted cutoff for risk reclassification and initiation of preventive therapy [11].

The study was conducted in accordance with the principles of the Declaration of Helsinki and received approval from the institutional ethics committee (TABED 2-25-1702, on 26 November 2025). Because of the retrospective design and reliance on existing medical records, the requirement for informed consent was waived.

### Statistical Analysis

Data analysis was performed using the SPSS 18 Statistical Package Program for Windows (SPSS Inc., Chicago, IL, USA). Continuous variables are presented as mean ± standard deviation (SD) or median (25th percentile–75th percentile) based on their distribution, and categorical variables as counts (percentages). The normality of the data was assessed using the Kolmogorov–Smirnov test. Comparisons between CACS groups (CACS < 100 vs. CACS ≥ 100) were conducted using the Independent Samples T-test or Mann–Whitney U test for continuous variables and the Chi-square test for categorical variables.

Multivariate Binomial Logistic Regression Analysis was performed to determine whether Whole Blood Viscosity (WBV) is independently associated with a high CACS (≥100). To ensure the robustness of our findings and avoid information loss associated with dichotomization, we conducted several sensitivity analyses. The relationship between WBV and CACS was evaluated as continuous variables using Spearman correlation analysis. The correlation analysis was repeated by excluding patients with a CACS of 0, assessing the association specifically in the presence of detectable calcification. The potential effect modification by sex and diabetes status was explored by adding interaction terms (WBV × Sex and WBV × Diabetes) into the multivariate models. Statistical significance was set at a two-tailed *p*-value of < 0.05

## 3. Results

A total of 403 patients who underwent CCTA were included in the final analysis. The patients were categorized into two groups according to the widely accepted threshold for coronary artery calcium score of CACS < 100 (n = 258) and CACS ≥ 100 (n = 145). The median Agatston score was 0 (IQR: 0–100) in the CACS < 100 group and 300 (IQR: 180–535) in the CACS ≥ 100 group. Table 1 summarizes the baseline characteristics of the study population.

Patients in the CACS ≥ 100 group were significantly older (61 ± 10 vs. 48 ± 10 years; *p* < 0.001), had a lower proportion of females (29% vs. 49%; *p* = 0.001), and demonstrated a notably higher prevalence of diabetes mellitus (31.6% vs. 15.3%; *p* < 0.001) and hypertension (53.1% vs. 29.8%; *p* < 0.001) compared with those in the CACS < 100 group. The CACS ≥ 100 group also exhibited significantly higher fasting glucose, creatinine levels, and indicators of left ventricular hypertrophy, including increased interventricular septal thickness and posterior wall thickness. Despite marked differences in traditional cardiovascular risk factors, there was no statistically significant difference in calculated WBV values between the two CACS groups. Mean WBV at high shear rate (HSR, 208/s) was 4.3 ± 0.5 cP in the CACS < 100 group and 4.4 ± 0.5 cP in the CACS ≥ 100 group (*p* = 0.456). Similarly, mean WBV at low shear rate (LSR, 0.5/s) was 29.9 ± 8.0 cP and 30.4 ± 8.6 cP, respectively (*p* = 0.505). This absence of association remained unchanged after adjusting for age and sex, with adjusted *p*-values of 0.249 for HSR and 0.282 for LSR, see Table 2.

To avoid information loss associated with dichotomization, we further evaluated the relationship between WBV and CACS as continuous variables. Spearman correlation analysis revealed no significant association between continuous CACS and either WBV at High Shear Rate, 208/s (r = 0.054, *p* = 0.280) or WBV at Low Shear Rate, 0.5/s (r = 0.05, *p* = 0.317). Additionally, to address the potential impact of patients with a CACS of 0 (n = 190), we performed a subgroup analysis excluding the patients with a CACS of 0. Even in the presence of detectable coronary calcification (CACS > 0), no significant correlation was observed between WBV and the extent of calcium burden (WBV at High Shear Rate, 208/s r = −0.084; *p* = 0.221, and WBV at Low Shear Rate, 0.5/s r = −0.082; *p* = 0.236).

Two separate multivariate binomial logistic regression models were constructed to identify independent predictors of CACS ≥ 100 (Table 3). In Model 1, after adjustment for age, sex, smoking status, diabetes mellitus, and hypertension, WBV at LSR was not independently associated with high CACS (OR: 0.987, 95% CI: 0.949–1.027; *p* = 0.520). Similarly, in model 2, WBV at HSR also failed to demonstrate an independent association (OR: 0.489, 95% CI: 0.419–1.485; *p* = 0.462). Consistent with the univariate results, age (OR: 1.158 per year; *p* < 0.001) and sex (OR: 3.932; *p* < 0.001 in Model 2) remained the strongest independent predictors of belonging to the CACS ≥ 100 group.

We explored potential effect modifications by key clinical strata, including sex and diabetes mellitus. In our multivariate logistic regression models, we included interaction terms (e.g., WBV × Sex and WBV × Diabetes mellitus). None of these interaction terms reached statistical significance (for WBV at LSR × Sex category and WBV at HSR × Sex category, *p*-interaction = 0.092, for WBV at LSR × Diabetes category, p-interaction = 0.245 and for WBV at HSR x Diabetes category, *p*-interaction = 0.250), indicating that the lack of association between WBV and high CACS was consistent across these subgroups. Furthermore, subgroup-specific multivariate analyses confirmed that WBV was not an independent predictor of high CACS in either males or females, or in diabetic vs. non-diabetic patients.

## 4. Discussion

This study investigates whether WBV, calculated using the De Simone formula, is independently associated with the presence of significant coronary calcification, defined as a CACS of 100 or higher. Contrary to theoretical expectations based on hemorheology and atherogenesis, our principal finding is the lack of a significant association between calculated WBV (at both high and low shear rates) and high CACS. WBV values were statistically similar in the CACS < 100 and CACS ≥ 100 groups. Furthermore, in multivariate binomial logistic regression models adjusted for traditional cardiovascular risk factors (age, sex, diabetes mellitus, hypertension, and smoking status), neither WBV at high shear rate nor WBV at low shear rate emerged as an independent predictor of a high CACS.

Our decision to use the CACS ≥ 100 threshold was primarily a clinically driven choice, as this level marks a critical intervention point for statin and aspirin therapy according to the National Lipid Association, Society of Cardiovascular Computed Tomography (guidelines [11]. However, to ensure methodological rigor, we also tested CACS as a continuous variable. The lack of a significant correlation in both categorical and continuous models suggests that calculated WBV may not have a direct linear or threshold-based association with the calcific phenotype of atherosclerosis.

Previous clinical studies have demonstrated an association between calculated WBV and various aspects of CAD. Elevated WBV has been linked to higher SYNTAX scores reflecting more complex and extensive coronary lesions as well as to lower Fractional Flow Reserve (FFR), indicating greater functional significance [12,13]. Collectively, these findings suggest that WBV may influence coronary flow resistance and overall plaque burden. Elevated WBV has also been reported to correlate with other manifestations of atherosclerosis and related complications [14,15]. At first glance, the results of our study may appear inconsistent with these earlier observations. However, it is important to recognize that Coronary Artery Calcium (CAC) represents intraplaque calcification, which arises relatively late in the natural history of atherosclerotic plaque development. Plaque progression encompasses initiation, growth, stabilization, and ultimately calcification. Our findings, therefore, raise the possibility that WBV may affect the total plaque burden, particularly the non-calcified components implicated in plaque vulnerability [9,10] without necessarily influencing the degree of calcification reflected by CAC. In practical terms, higher WBV may promote the formation or progression of non-calcified (soft) plaque, the substrate most closely associated with acute coronary syndromes, rather than accelerating the calcification process itself. Supporting this interpretation, Lee et al. found that coronary whole blood viscosity was higher in the acute coronary syndrome group than in the non-acute coronary syndrome group [16]. It is important to consider that WBV may have a more significant impact on the earlier stages of atherosclerosis, such as the development of non-calcified or ‘soft’ vulnerable plaques, rather than the late-stage calcific phenotype represented by CACS. High viscosity-induced wall shear stress is known to promote endothelial dysfunction and lipid accumulation, which are hallmarks of early-stage plaque formation. Since our study focused solely on CACS, the potential association between WBV and non-calcified plaque burden remains to be elucidated. Future investigations using intravascular imaging modalities such as intravascular ultrasound (IVUS) and optical coherence tomography (OCT) may help clarify the relationship between WBV, plaque composition, and calcification dynamics [17].

The absence of an independent association between WBV and a high coronary artery calcium score (CAC) in our study is consistent with findings reported in the literature [18]. In a cohort of patients undergoing coronary angiography, Vosseler et al. similarly did not observe a significant correlation between hemorheological parameters, including blood viscosity, and the extent of coronary or carotid atherosclerosis. They also found no apparent relationship with endothelial function, which led them to suggest that hemorheological assessments may be limited in their utility for evaluating subclinical or established atherosclerosis in routine clinical practice. The alignment between our CAC-based results and Vosseler’s angiography-based findings suggests that WBV may lack the sensitivity required to reliably reflect the severity of established plaque burden.

The lack of association between WBV and CACS in our study contrasts with previous reports linking elevated WBV to other cardiac calcifications, such as Mitral Annular Calcification (MAC) and Aortic Valve Sclerosis (AVS) [6,7]. While our study did not directly assess MAC or AVS, this divergence in the literature may reflect the influence of different local hemodynamic environments on calcification processes. It has been suggested that in regions characterized by lower shear stress or mechanical strain, such as the mitral annulus, WBV might play a more prominent role in promoting local stasis and inflammation [19,20,21]. Conversely, in the high-flow environment of the coronary arteries, the transition to a late-stage calcific phenotype (CACS) appears to be driven more dominantly by traditional risk factors rather than estimated rheological parameters. Our findings, consistent with some previous observations [18], suggest that the clinical utility of calculated WBV may be more pronounced in specific hemodynamic contexts rather than as a general predictor of coronary calcium burden. Further comparative studies involving multi-site calcification assessments are needed to confirm these potential regional differences.

Our study has several limitations that merit consideration. First, WBV values were estimated using the De Simone formula rather than being measured directly with rheometry. Although this method is practical, it lacks the sensitivity required to capture complex rheological properties such as red blood cell aggregation and deformability that may influence vascular physiology. Second, because CACS quantifies only the calcified (and thus more stable) portion of coronary plaque, it does not account for non-calcified, vulnerable plaque. This prevents us from determining whether WBV is associated with total plaque burden or soft plaque components that are more relevant to acute coronary syndromes. Our study relied on CACS and did not include CCTA data for plaque characterization. Therefore, we were unable to assess the relationship between WBV and high-risk plaque features or non-calcified plaque volume. Third, the cross-sectional nature of the study limits causal inference, underscoring the need for longitudinal investigations to assess whether WBV influences the progression of coronary calcification over time. Excluding conditions such as acute blood loss, chronic kidney or liver disease, paraproteinemia, hematologic disorders, or anemia (which significantly affect WBV) may limit the generalizability of our findings to these specific patient populations. Finally, the high proportion of participants with CACS = 0 may have reduced statistical power, potentially obscuring subtle associations. Future studies should incorporate direct rheological assessment and utilize advanced imaging modalities such as intravascular ultrasound (IVUS) or optical coherence tomography (OCT) to examine WBV’s relationship with specific plaque morphologies, including soft versus calcified plaque, in order to better define its role across the full spectrum of atherosclerosis.

## 5. Conclusions

In conclusion, this study found no independent or significant association between calculated Whole Blood Viscosity (WBV) and high-risk coronary calcification (CACS ≥ 100), even after adjustment for traditional cardiovascular risk factors. Our findings, therefore, suggest that WBV, when derived from routine clinical parameters, does not provide additional predictive value for identifying patients with substantial coronary calcification beyond established risk factors. Future studies incorporating direct rheological assessment and advanced plaque imaging modalities will be essential to clarify the specific role of WBV across different stages and morphologies of atherosclerosis.

## Figures and Tables

**Table 1:** Comparison of demographic, clinical, and laboratory parameters across coronary calcification score groups.

	CAC Score < 100 n = 258	CAC Score ≥ 100 n = 145	*p*
Age, years	48 ± 10	61 ± 10	<0.001
Sex, female, (%)	125 (49)	42 (29)	0.001
Smokers, (%)	89 (34.9)	58 (42.6)	0.082
Diabetes mellitus, (%)	39 (15.3)	43 (31.6)	<0.001
Hypertension, (%)	74 (29.8)	68 (53.1)	<0.001
Glucose, mg/dL	100 ± 33	110 ± 41	0.007
Creatinine, mg/dL	0.81 ± 0.17	0.86 ± 0.15	0.005
Sodium, mEq/L	140 ± 2	141 ± 2	0.070
Potassium, mEq/L	4.4 ± 0.3	4.3 ± 0.3	0.130
Total cholesterol, mg/dL	201 ± 43	204 ± 60	0.702
Triglyceride, mg/dL	148 (101–200)	148 (109–196)	0.921
High-Density Lipoprotein, mg/dL	46 ± 14	45 ± 11	0.408
Low-Density Lipoprotein, mg/dL	123± 35	127 ± 56	0.376
Leukocytes,/mm^3^	7.360 ±1.921	7.417 ± 1.930	0.773
Hemoglobin, g/dL	14.2 ± 1.5	14.4 ± 1.4	0.140
Hematocrit, %	43.1 ± 4.1	44.5 ± 4.3	0.348
Platelets,/mm^3^	263.166 ± 65.950	251.310 ± 59.460	0.074
Alanine Aminotransferase, U/L	25 (18–32)	22 (17–31)	0.147
Aspartate Aminotransferase, U/L	18 (14–24)	16 (13–22)	0.010
Albumin, g/dL	4.6 ± 0.3	4.5 ± 0.3	<0.001
Total protein, g/dL	7.1 ± 0.4	7.1 ± 0.3	0.224
**Echocardiography**			
Left ventricular ejection fraction, %	60 ± 4	59 ± 5	0.021
End diastolic diameter, mm	46 ± 4	46 ± 3	0.153
Interventricular septum thickness, mm	10.3 ± 1.3	10.9 ± 1.7	<0.001
Left ventricle posterior wall thickness, mm	9.9 ± 1.1	10.2 ± 1	0.005
**Whole blood viscosity**			
High Shear rate = 208/s	4.3 ± 0.5	4.4 ± 0.5	0.456
Low Shear rate = 0.5/s	29.9 ± 8.0	30.4 ± 8.6	0.505
**Coronary Calcium**			
Agatstone score	0 (0–100)	300 (180–535)	

**Table 2:** Age and sex adjusted whole blood viscosities according to coronary calcification score groups.

	CAC Score < 100 n = 258	CAC Score ≥ 100 n = 145	*p*	Age and Sex Adjusted *p*
WBV at High Shear Rate, 208/s	4.3 ± 0.5	4.4 ± 0.5	0.456	0.249
WBV at Low Shear Rate, 0.5/s	29.9 ± 8.0	30.4 ± 8.6	0.505	0.282

WBV: Whole blood viscosity.

**Table 3:** Univariate and multivariate logistic regression analysis for coronary calcium score groups.

	Univariate		Multivariate	
Model 1	OR (95% CI)	*p*	OR (95% CI)	*p*
Age, years	1.151 (1.117–1.186)	<0.001	1.158 (1.120–1.197)	<0.001
Sex	2.119 (1.1383–3.246)	0.001	3.855 (1.933–7.688)	<0.001
Smokers	1.387 (0.905–2.124)	0.133	1.615 (0.911–2.863)	0.101
Diabetes mellitus	2.697 (1.664–4.372)	<0.001	1.746 (0.904–3.373)	0.097
Hypertension, (%)	2.662 (1.747–4.056)	<0.001	1.379 (0.765–2.486)	0.285
WBV at Low Shear Rate, 0.5/s	1.008 (0.984–1.034)	0.505	0.987 (0.949–1.027)	0.520
**Model 2**	**OR (95% CI)**	***p***	**OR (95% CI)**	***p***
Age, years	1.151 (1.117–1.186)	<0.001	1.158 (1.120–1.197)	<0.001
Sex	2.119 (1.1383–3.246)	0.001	3.932 (1.961–7.883)	<0.001
Smokers	1.387 (0.905–2.124)	0.133	1.619 (0.913–2.872)	0.099
Diabetes mellitus	2.697 (1.664–4.372)	<0.001	1.741 (0.901–3.365)	0.099
Hypertension, (%)	2.662 (1.747–4.056)	<0.001	1.379 (0.765–2.485)	0.286
WBV at High Shear Rate, 208/s	1.163 (0.783–1.728)	0.455	0.489 (0.419–1.485)	0.462

WBV: Whole blood viscosity.

## Data Availability

The data presented in this study are available upon request from the corresponding author.
